# Virus Elimination by Direct-Acting Antiviral Agents Impacts Glucose Homeostasis in Chronic Hepatitis C Patients

**DOI:** 10.3389/fendo.2021.799382

**Published:** 2022-01-13

**Authors:** Chun-Han Cheng, Chia-Ying Chu, Huan-Lin Chen, I-Tsung Lin, Chia-Hsien Wu, Yuan-Kai Lee, Ming-Jong Bair

**Affiliations:** ^1^ Division of Gastroenterology, Department of Internal Medicine, Taitung Mackay Memorial Hospital, Taitung, Taiwan; ^2^ Department of Medicine, Mackay Medical College, New Taipei, Taiwan; ^3^ Department of Pathology, Taitung Mackay Memorial Hospital, Taitung, Taiwan

**Keywords:** direct-acting antiviral agents, genotype, hepatitis C, homeostasis model assessment, insulin resistance, type 2 dabetes

## Abstract

**Background and Aims:**

Chronic hepatitis C virus (HCV) infection is associated with dysregulation of glucose homeostasis, including insulin resistance (IR) and type 2 diabetes. However, independent risk factors associated with IR in chronic HCV-infected patients have not been detailly elucidated. Previous data regarding the impact of HCV elimination by direct-acting antiviral agents (DAAs) on glucose homeostasis is insufficient and controversial. This study aimed to analyze the independent factors associated with IR and to evaluate the changes in glucose homeostasis in chronic HCV-infected patients treated with DAAs therapies.

**Methods:**

We screened 704 patients with chronic HCV infection who underwent treatment with interferon-free DAAs. Patients’ baseline characteristics, biochemical and virological data were collected. The outcome measurements were their IR and β-cell function assessed by the homeostasis model assessment (HOMA) method at baseline and 12-weeks post-treatment.

**Results:**

High IR (HOMA-IR ≥ 2.5) was observed in 35.1% of the patients. Multivariable logistic regression analysis revealed that body mass index (BMI) >25 kg/m^2^, treatment experience, elevated baseline levels of alanine aminotransferase (ALT) and triglyceride, as well as Fibrosis-4 score >3.25 were independently associated with high IR. In patients who achieved sustained virological response (SVR), no significant change in mean HOMA-IR was observed from baseline to 12-weeks post-treatment (2.74 ± 2.78 to 2.54 ± 2.20, *p* = 0.128). We observed a significant improvement in β-cell secretion stress from 121.0 ± 110.1 to 107.6 ± 93.0 (*p* = 0.015). Subgroup analysis revealed that SVR was associated with a significant reduction in mean HOMA-IR in patients with baseline HOMA-IR ≥ 2.5 (5.31 ± 3.39 to 3.68 ± 2.57, *p* < 0.001), HCV genotype 1 (3.05 ± 3.11 to 2.62 ± 2.05, *p* = 0.027), and treatment experience (4.00 ± 3.37 to 3.01 ± 2.49, *p* = 0.039).

**Conclusions:**

There were several independent factors associated with IR in patients with chronic HCV infection, including obesity, treatment experience, high serum ALT and triglyceride levels, as well as advanced hepatic fibrosis. After viral elimination by DAAs, we observed a significant reduction in mean HOMA-IR in patients with baseline high IR, HCV genotype 1, and treatment experience.

## Introduction

Chronic hepatitis C virus (HCV) infection is a major cause of chronic hepatitis, cirrhosis, and hepatocellular carcinoma. It is also associated with multiple extrahepatic complications that influence the clinical outcomes of patients. An important extrahepatic manifestation is abnormal glucose metabolism including insulin resistance (IR), β-cell dysfunction, and diabetes mellitus (DM) (1–3). A prospective study showed that patients with chronic HCV infection were more than 11 times as likely as those without HCV infection to develop diabetes (4). A meta-analysis revealed a 1.8-fold higher risk of type 2 DM in HCV-infected patients than in hepatitis B virus (HBV)-positive/HCV-negative patients (5). Additionally, glucose abnormalities can worsen hepatic outcomes in HCV-infected patients (6). IR promotes the progression of hepatic fibrosis. This may be due to the direct effect of insulin on the proliferation of hepatic stellate cells and secretion of extracellular matrix (7, 8). Further, high glucose levels and hyperinsulinemia can lead to upregulation of connective growth factor that is involved in the pathogenesis of liver fibrosis (9). In HCV-related chronic liver disease, type 2 diabetes and IR are independently associated with rapid progression of liver fibrosis and increased risk of hepatocellular carcinoma (10, 11), as well as the elevated rate of hepatic morbidity and mortality (12).

The mechanisms underlying HCV-induced IR and DM are complex and poorly explained. The HCV genome is composed of structural (core, E1, and E2) and nonstructural (NS2-NS5B) genes. The complex effects of HCV core and nonstructural 5A (NS5A) proteins have been observed to play important roles in glucose metabolism (13). In normal circumstances, when insulin attaches to the hepatocyte receptor, the insulin receptor substrate (IRS) is phosphorylated and then causes activation of Akt. The activated Akt causes the translocation of glucose transporter-4 to the surface of the hepatocyte and facilitates glucose entry into the hepatocyte. Akt also induces the synthesis of glycogen and inhibition of hepatic gluconeogenesis (14). The HCV core protein impairs insulin signaling *via* several post-receptor mechanisms, including the activation of suppressor of cytokine signaling (SOCS) family members and consequent decrease in IRS-1 (15). The core protein also increases phosphorylation of IRS-1 at serine rather than tyrosine in hepatocytes, again preventing the downstream activation of Akt (14). HCV NS5A upregulates protein phosphatase 2A and inactivates Akt, which reduces the expression of glucose transporters GLUT1 and GLUT2, leading to a reduction in glucose uptake in the hepatocytes, and thus, induces IR (16). Additionally, HCV protein increases oxidative stress and mitochondrial dysfunction and leads to overexpression of inflammatory cytokines, such as tumor necrosis factor alpha, interleukin (IL)-6, and IL-8, resulting in systemic effect including hyperinsulinemia, hypertriglycemia, and down-regulation of adiponectin (17). Epidemiological data suggest that all the major HCV genotypes lead to IR. Some viral-specific factors that influence glucose metabolism have also been discussed. Molecular evidence suggests that HCV core proteins in genotypes 3a and 1b promote IRS-1 degradation *via* different mechanisms (18). IR was clinically found to be associated with HCV genotype 1 and a high viral load, although the results were inconsistent (4, 19, 20).

Elimination of HCV by interferon (IFN)-based regimen may perturb glucose homeostasis. Previous observational studies have shown that successful eradication of HCV improves IR in patients receiving IFN-based therapy (20, 21). The β-cell hyperfunction is also ameliorated after antiviral treatment (22). Therefore, elimination or suppression of HCV can reduce the incidence of type 2 diabetes (23, 24). However, a few studies revealed no change in fasting glucose, insulin, and homeostatic model assessment-IR (HOMA-IR) after antiviral treatment (25, 26). The results of these studies are inconsistent, but they indicate that the benefits of glucose metabolism may also be HCV genotype specific.

Treatment has dramatically improved in the era of direct-acting antiviral agents (DAAs). These modern drugs have extremely high treatment efficacy, safety, and less adverse events. These new medications have a higher rate of viral clearance than IFN-based therapy but lack the direct effect of IFN on glucose metabolism. Moreover, the impact of HCV clearance by DAAs on glucose abnormalities remains to be evaluated.

Given the presumption that chronic HCV infection involves the development of IR, we hypothesized that HCV eradication by DAAs treatment could improve IR. The aim of the present study was to explore potential factors associated with IR in chronic HCV-infected patients and to assess the impact of HCV elimination on glucose homeostasis in patients who received DAAs therapies.

## Methods

### Study Design

This was a single-center, observational study. Chronic HCV-infected patients with DAAs treatment were enrolled. Laboratory data including glucose parameters at baseline and 12-weeks post-treatment were measured. Several factors associated with baseline high IR were analyzed. Further, we calculated the dynamic changes in IR and β-cell secretion function in patients whose viruses were successfully eradicated. This study was conducted in accordance with the principles of the Declaration of Helsinki, and the study design was approved by the Institutional Review Board of the Mackay Memorial Hospital. Informed consent was obtained from the study participants.

### Patients

We enrolled patients with chronic HCV infection who underwent treatment with IFN-free DAAs therapy between January 2017 and July 2020. The inclusion criteria were patients (1) aged ≥ 20 years; (2) with detectable HCV viral load in the blood prior to treatment; (3) who agreed to receive laboratory testing at baseline, during antiviral treatment, and at 12 weeks after treatment; and (4) who provided informed consent. Patients were excluded if they: (1) missed regular clinical follow-ups after treatment; (2) had a medical history of DM or used glucose-lowering drugs; (3) died from other etiology during the study period; and (4) had concurrent conditions, including human immunodeficiency virus or HBV coinfection, Wilson’s disease, primary biliary cirrhosis, hemochromatosis, and autoimmune hepatitis. Basic patient information, body mass index (BMI), HCV viral loads and genotype, alcohol consumption, and medical history were collected. Patients received different DAAs regimens, including asunaprevir + daclatasvir (2.5%), paritaprevir + ritonavir + ombitasvir + dasabuvir (7.8%), elbasvir + grazoprevir (10.7%), glecaprevir + pibrentasvir (16.6%), and sofosbuvir-based regimens (62.4%). Each patient received one of these DAAs regimens based on the HCV genotype, previous treatment status, presence of cirrhosis, current medications, and comorbidities.

### Laboratory Examinations

Infection with chronic HCV was defined as persistent viremia for at least six months before treatment. Serum HCV ribonucleic acid (RNA) levels were quantified using a Roche Amplicor polymerase chain reaction (PCR) assay, in which the lowest level of detection was 15 IU/mL. HCV genotyping was performed using primer-specific PCR and direct PCR deep sequencing with an ABI 3730 sequencer. The sustained virological response (SVR) was defined as undetectable serum HCV RNA levels 12 weeks following treatment cessation. Information on the levels of fasting glucose, insulin, aspartate transaminase (AST), alanine aminotransferase (ALT), bilirubin, albumin, cholesterol, triglyceride, alpha-fetoprotein, white blood cells (WBC), as well as hemoglobin and platelets at baseline and 12-weeks post-treatment were collected. We used Fibrosis-4 (FIB-4) index as the non-invasive measurement for hepatic fibrosis. The FIB-4 index was calculated using the following formula: (AST [IU/L] × age [years])/(platelet count [10^9^/L] × ALT [IU/L]^1/2^).

The glucose homeostasis was assessed by the HOMA method. The formulas for the HOMA model are as follows: HOMA-IR = fasting glucose (mg/dL) × fasting insulin level (μU/mL)/405; HOMA-β = fasting insulin level (μU/mL) × 360/(fasting glucose [mg/dL]–63). We dichotomized patients at a cutoff point of HOMA-IR 2.5 and analyzed factors associated with IR.

### Statistical Analysis

Categorical data are presented as numbers (percentages) and continuous variables are presented as mean ± standard deviation. The significance of the difference between categorical variables was determined using the Chi-square test or Fisher’s exact test depending on the sample size; continuous variables were compared using Student’s *t*-test. For continuous variables with wide distribution, we presented data as median (interquartile range) and applied the Mann-Whitney test to evaluate the differences. After univariate analysis, we performed multivariable logistic regression modelling to identify factors that were independently associated with baseline high IR. To compare quantitative glucose parameters between baseline and post-treatment, we performed Paired *t*-test. All statistical analyses were defined as two-sided hypotheses with a significance level defined at *p* < 0.05. Statistical analyses were performed using SPSS 22.0 (SPSS Inc., Chicago, IL, USA).

## Results

### Baseline Characteristics

During the study period, 704 patients were treated with IFN-free DAAs. We excluded 192 patients with medical history of DM or who used glucose-lowering drugs. Additionally, 65 patients were excluded from the study due to failure of checking insulin levels at either baseline or follow-up (n=34), missing regular follow-up (n=24), and death during treatment (n=7). Finally, we enrolled 447 patients in the study. The baseline characteristics, laboratory results, glucose parameters, DAAs regimens, and treatment responses are shown in **Table 1**. The patients were predominantly women (52.8%), with a mean BMI of 25.5 ± 4.5 kg/m^2^, which was in the upper range of normal weight. The most frequent HCV genotype was genotype 1 (53.7%), followed by genotype 2 (35.1%). The prevalence of high viral loads (> 80 × 10^4^ IU/mL) was 67.3%. Sofosbuvir-based regimens were most commonly used, accounting for 62.4%.

**Table 1:** Baseline characteristics (N = 447).

Male gender	211 (47.2)
Age (mean ± SD)	60.1 ± 13.1
Indigenous race	138 (30.1)
BMI (kg/m^2^, mean ± SD)	25.5 ± 4.5
Alcoholism	34 (7.6)
^a^Baseline HCV viral load (10^4^ IU/mL, median (IQR))	238.9 (506.3)
Baseline HCV viral load > 80 x 10^4^ IU/mL	301 (67.3)
HCV genotype 1	240 (53.7)
Treatment experience	45 (10.1)
Antiviral regiments	
Asunaprevir + Daclatasvir	11 (2.5)
Paritaprevir + Ritonavir + Ombitasvir + Dasabuvir	35 (7.8)
Elbasvir + Grazoprevir	48 (10.7)
Glecaprevir + Pibrentasvir	74 (16.6)
Sofosbuvir-based regiments	279 (62.4)
^a^AST (IU/L, median (IQR))	44.0 (44.0)
^a^ALT (IU/L, median (IQR))	47.0 (54.0)
Total bilirubin (mg/dL, mean ± SD)	0.9 ± 0.4
Albumin (g/dL, mean ± SD)	4.1 ± 0.4
^a^Alpha-fetoprotein (ng/mL, median (IQR))	3.8 (4.4)
Triglyceride (mg/dL, mean ± SD)	98.7 ± 57.5
Cholesterol (mg/dL, mean ± SD)	171.7 ± 35.6
WBC (10^3^/µL, mean ± SD)	5480 ± 1635
FIB-4 (median (IQR))	2.08 (2.35)
FIB-4 > 3.25	130 (29.1)
HOMA-IR (mean ± SD) (median (IQR))	2.73 ± 2.77 [1.83 (2.10)]
HOMA-IR ≥ 2.5	157 (35.1)
HOMA-β (mean ± SD) (median (IQR))	120.6 ± 109.4 [88.7 (85.7)]
SVR	438 (98.0)

Categorical data are presented as number (percentage).

a These continuous variables showed wide distribution. They were presented with median (interquartile range).

ALT, alanine aminotransferase; AST, aspartate transaminase; BMI, Body mass index; FIB-4, Fibrosis-4; HCV, hepatitis C virus; HOMA, homeostasis model assessment; IR, insulin resistance; IQR, interquartile range; SD, standard deviation; SVR, sustained virological response; WBC, white blood cell.

### Variables Associated With Baseline IR

At baseline, 157 patients (35.1%) presented with high IR (HOMA-IR ≥ 2.5). Univariate analysis (**Table 2**) showed that several factors were significantly associated with high IR, including indigenous race, high BMI (> 25 kg/m^2^), alcoholism, baseline elevated levels of ALT, alpha-fetoprotein, and triglyceride, as well as hepatic fibrosis (defined as FIB-4 > 3.25). The association between IR and older age was not significant (*p*=0.073). For virus-specific parameters, HCV genotype 1 and HCV treatment experience were significantly associated with high IR (both *p* < 0.01). The baseline HCV viral load did not influence IR in our patients (*p*=0.953). Baseline bilirubin, creatinine, uric acid, WBC counts, hemoglobin, and low-density lipoprotein (LDL) were not significantly associated with baseline high IR.

**Table 2:** Univariate analysis of baseline factors associated with HOMA-IR.

Factors	HOMA-IR ≥ 2.5 (n = 157)	HOMA-IR < 2.5 (n = 290)	*p* value
Male gender	68 (43.3)	89 (56.7)	0.225
Age (mean ± SD)	61.6 ± 12.2	59.3 ± 13.5	0.073
Indigenous race	59 (37.6)	82 (28.3)	**0.043**
BMI (kg/m^2^, mean ± SD)	28.6 ± 5.0	24.3 ± 3.7	**0.000**
BMI > 25	102 (65.8)	106 (37.2)	**0.000**
Alcoholism	18 (11.5)	16 (5.5)	**0.024**
Baseline HCV viral load > 80 x 10^4^ IU/mL	106 (67.5)	195 (67.2)	0.953
HCV genotype 1	99 (63.1)	141 (48.6)	**0.003**
Treatment experience	27 (17.2)	18 (6.2)	**0.000**
^a^ALT (IU/L, median (IQR))	60.0 (64.5)	39.5 (56.0)	**0.000**
^a^Alpha-fetoprotein (ng/mL, median (IQR))	5.2 (7.5)	3.2 (3.0)	**0.000**
Triglyceride (mg/dL, mean ± SD)	113.6 ± 72.6	90.6 ± 45.6	**0.000**
Cholesterol (mg/dL, mean ± SD)	168.8 ± 37.1	173.2 ± 34.8	0.261
WBC (10^3^/µL, mean ± SD)	5586 ± 1660	5422 ± 1620	0.312
FIB-4 > 3.25	63 (40.1)	67 (23.1)	**0.000**

Categorical data are presented as number (percentage).

a These continuous variables showed wide distribution. They were presented with median (interquartile range).

ALT, alanine aminotransferase; AST, aspartate transaminase; BMI, Body mass index; FIB-4, Fibrosis-4; HCV, hepatitis C virus; HOMA, homeostasis model assessment; IR, insulin resistance; IQR, interquartile range; SD, standard deviation; WBC, white blood cell.

The bold fonts means a p value less than 0.05.

Further, we performed multivariable logistic regression analysis (**Table 3**), which revealed that BMI > 25 kg/m^2^, treatment experience, baseline elevated ALT and triglyceride levels, as well as FIB-4 > 3.25 were independently associated with high IR (all *p* < 0.05). After adjustment of other relevant variables, some factors showed insignificant associations, including race, HCV genotype, alcoholism, and serum alpha-fetoprotein levels.

**Table 3:** Multivariable logistic regression analysis for factors associated with high insulin resistance.

Factors	OR (95% CI)	*p* value
Indigenous race	1.307 (0.812 – 2.106)	0.270
BMI > 25	2.685 (1.709 – 4.219)	**0.000**
Alcoholism	1.886 (0.841 – 4.232)	0.124
HCV genotype 1	1.556 (0.978 – 2.475)	0.062
Treatment experience	3.379 (1.635 – 6.984)	**0.001**
Elevated ALT	2.034 (1.263 – 3.277)	**0.004**
Elevated alpha-fetoprotein	1.300 (0.801 – 2.109)	0.288
Elevated triglyceride	2.366 (1.498 – 3.736)	**0.000**
FIB-4 > 3.25	1.751 (1.057 – 2.902)	**0.030**

Laboratory examinations were dichotomized at median value.

ALT, alanine aminotransferase; BMI, Body mass index; CI, Confidence interval; FIB-4, Fibrosis-4; OR, Odds ratio.

The bold fonts means a p value less than 0.05.

### Outcome of Viral Elimination on Glucose Parameters

**Figure 1:**
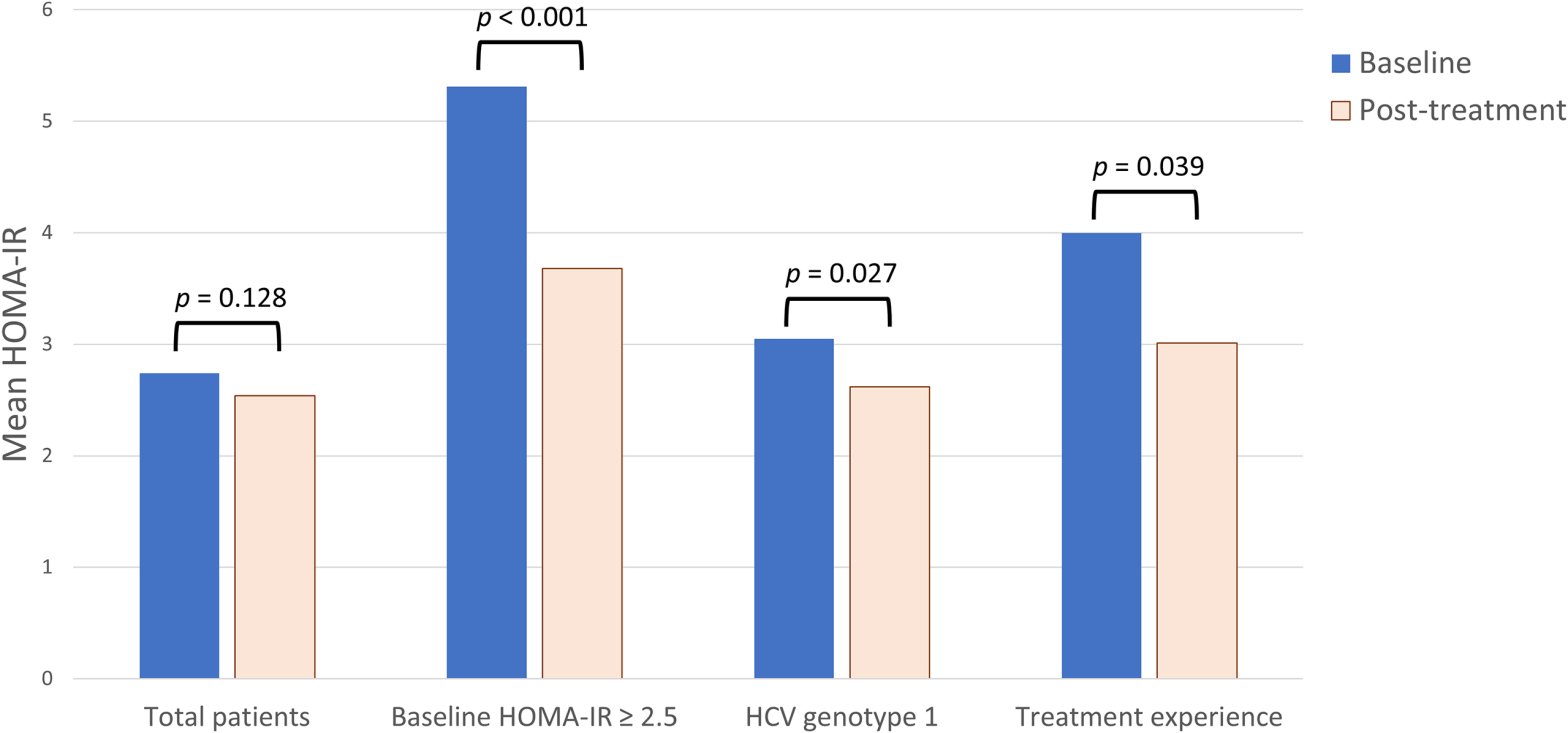
The change of insulin resistance (IR) in hepatitis C virus (HCV)-infected patients treated with direct-acting antiviral agents. Homeostasis model assessment (HOMA)-IR at baseline and 12-weeks after antiviral treatment were measured. In general, there was no significant change in mean HOMA-IR. In subgroup analysis, IR significantly reduced after antiviral treatment in patients with baseline HOMA-IR ≥ 2.5, HCV genotype 1, and treatment experience.

Of 447 patients, 438 (98.0%) achieved SVR. To analyze the relationship between viral elimination and changes in glucose parameters, HOMA-IR and HOMA-β were considered as continuous variables (**Table 4**). HOMA-IR did not change significantly from baseline to 12-weeks after treatment (2.74 ± 2.78 to 2.54 ± 2.20, *p* = 0.128). There was a significant improvement in β-cell secretion stress (121.0 ± 110.1 to 107.6 ± 93.0, *p* = 0.015). In patients with baseline HOMA-IR ≥ 2.5, we observed a significant improvement in mean IR (5.31 ± 3.39 to 3.68 ± 2.57, *p* < 0.001). Additionally, the subgroup analysis revealed that a statistically significant reduction in mean IR after DAAs therapies was observed in patients with HCV genotype 1 (3.05 ± 3.11 to 2.62 ± 2.05, *p* = 0.027) and treatment experience (4.00 ± 3.37 to 3.01 ± 2.49, *p* = 0.039) (**Figure 1**). However, in patients with baseline HOMA-IR < 2.5, there was a significant increase in mean IR after HCV eradication. We also analyzed pre- and post-treatment HOMA-IR in patients with different races, BMI, HCV viral loads, baseline laboratory results, and FIB-4 score. There was no significant difference in HOMA-IR between values at baseline and 12-weeks post-treatment.

**Table 4:** The change of glucose parameters among patients with SVR.

Patients	HOMA-IR			HOMA-β		
	Baseline	Post-treatment	*p* value	Baseline	Post-treatment	*p* value
Total patients (n = 438)	2.74 ± 2.78	2.54 ± 2.20	0.128	121.0 ± 110.1	107.6 ± 93.0	**0.015**
Baseline HOMA-IR ≥ 2.5 (n = 153)	5.31 ± 3.39	3.68 ± 2.57	**0.000**	190.5 ± 127.6	141.5 ± 116.1	**0.000**
< 2.5 (n = 285)	1.36 ± 0.59	1.93 ± 1.69	**0.000**	83.7 ± 77.0	89.3 ± 71.6	0.306
BMI > 25 (n = 204)	3.49 ± 3.45	3.20 ± 2.48	0.205	153.7 ± 136.2	133.2 ± 104.3	**0.049**
BMI < 25 (n = 227)	2.06 ± 1.77	1.96 ± 1.72	0.445	92.0 ± 68.3	85.0 ± 74.9	0.168
HCV genotype 1 (n = 236)	3.05 ± 3.11	2.62 ± 2.05	**0.027**	121.2 ± 112.8	105.8 ± 89.6	**0.045**
Genotype non-1 (n = 202)	2.37 ± 2.31	2.44 ± 2.37	0.684	120.7 ± 107.2	109.6 ± 96.9	0.163
Treatment experience (n = 41)	4.00 ± 3.37	3.01 ± 2.49	**0.039**	161.5 ± 133.7	152.9 ± 178.9	0.761
Treatment naïve (n = 397)	2.61 ± 2.69	2.48 ± 2.17	0.371	116.8 ± 106.7	102.9 ± 77.9	**0.010**
Baseline high ALT (n = 219)	3.29 ± 3.31	2.89 ± 2.41	0.064	130.0 ± 112.5	121.7 ± 112.2	0.336
Baseline high triglyceride (n = 219)	3.24 ± 3.10	2.86 ± 2.20	0.056	137.1 ± 119.2	115.2 ± 83.4	**0.006**
FIB-4 > 3.25 (n = 125)	3.79 ± 4.08	3.27 ± 2.94	0.137	139.3 ± 123.1	123.9 ± 122.6	0.210
FIB-4 < 3.25 (n = 313)	2.32 ± 1.91	2.25 ± 1.75	0.554	113.7 ± 103.8	101.0 ± 77.3	**0.035**

ALT, alanine aminotransferase; BMI, Body mass index; FIB-4, Fibrosis-4; HCV, hepatitis C virus; HOMA, homeostasis model assessment; IR, insulin resistance; SVR, sustained virological response.

The bold fonts means a p value less than 0.05.

## Discussion

We presented an observational analysis of glucose homeostasis in patients with chronic HCV infection and the outcomes of HCV elimination by DAAs therapy. We identified several factors contributing to IR in non-diabetic patients with chronic HCV infection. Obesity, elevated ALT and triglyceride levels, treatment experience, and advanced fibrosis were independently associated with IR. Obesity and metabolic syndrome were generally validated as risk factors for IR. In our result, serum levels of triglyceride, a well-known predictor of type 2 diabetes, were also independently related to IR. HCV NS5A and core proteins are known to directly inhibit microsomal triglyceride transfer protein activity. The accumulation of hepatic triglycerides reduces insulin-stimulated glycogen synthesis and enhances hepatic gluconeogenesis, consequently leading to hepatic and peripheral IR. We did not observe any virus-specific effects of IR. Neither HCV genotype nor viral loads were observed to be independent factors for HOMA-IR ≥ 2.5. At the molecular level, the core protein of genotype 3a promotes degradation of IRS-1 *via* downregulation of peroxisome proliferator-activated receptor gamma (PPAR-γ) and upregulation of SOCS-7; and the core protein of genotype 1b interferes with the insulin signaling by activating the mammalian target of rapamycin (18). Previous studies have investigated the association of HCV genotype 1 with IR, but the results were inconsistent (4, 20, 27, 28). In the present study, we also observed a higher prevalence of IR in patients with HCV genotype 1. However, the statistical power was attenuated in multivariable logistic regression analysis. This could be because that the genotype-specific mechanisms interfering with the insulin signaling pathway only partially influenced systemic IR. The established risk factors for diabetes, such as obesity and hyperlipidemia, remain the major contributors of IR in HCV-infected patients.

The multivariable logistic regression analysis in the present study demonstrated that treatment experience and advanced hepatic fibrosis were independently associated with IR. These findings were less discussed in previous articles. As explained earlier, the HCV core protein and NS5A have complex effects on the insulin signaling pathway, influence hepatic and systemic glucose homeostasis, and thus, induce IR. We hypothesized that prolonged inflammation resulting from chronic HCV infection is associated with dysregulation of glucose homeostasis. A previous history of treatment with IFN-based therapy may imply a longer duration of infection with HCV. Additionally, hepatic fibrosis indirectly supports the probability of a longer inflammation by HCV infection. In HCV-infected liver, inflammation is a key process to drive the fibrogenic response. The immune system secretes proinflammatory cytokines and profibrogenic factors, leading to hepatocellular injury, production of extracellular matrix, proliferation of myofibroblasts, and stimulation of fibrosis (29, 30). Our finding revealed an association between hepatic fibrosis and IR. It is noteworthy that our result only supported a correlation, not a causal relationship. Advanced liver fibrosis may impair insulin clearance, resulting in elevated serum insulin levels. On the contrary, IR also contributes to the progression of liver fibrosis. The mechanism could be a direct stimulation of liver stellate cells by hyperinsulinemia, resulting in increased production of the connective tissue growth factor and subsequent accumulation of extracellular matrix (9, 31).

We analyzed the outcome of glucose homeostasis in chronic HCV patients receiving DAAs. There was a significant improvement in β-cell stress. As mentioned earlier, levels of hepatic IRS1 and IRS2 decrease in HCV-infected patients. Clearance of HCV is accompanied by normalization of hepatic expression of IRS and improved β-cell function (21), which then influences glucose homeostasis. The IR remained statistically unchanged after DAAs therapy. This result is consistent with the results of previous studies that observed no difference in HOMA-IR between baseline and post-treatment phases in patients with HCV who underwent treatment (26, 32). However, this contradicts the hypothesis that IR may be improved after HCV eradication, especially as HCV infection is a risk factor for the development of diabetes. There may be several explanations for the lack of an association between SVR and improvement of IR. The development of IR in HCV-infected patients could be a combination of both host- and virus-mediated pathways. The complex hepatic and non-hepatic mechanisms affecting glucose homeostasis may lead to heterogeneous post-SVR outcomes regarding IR. We performed subgroup analysis for further elucidation of the possible causal relationship. We observed a significant improvement in IR in patients with baseline HOMA-IR ≥ 2.5, which indirectly supports that HCV is an important contributor to IR, especially among patients with high-IR status. Additionally, this high-IR status could be reversed after HCV elimination by DAAs. Our subgroup analysis suggested that elimination of HCV genotype 1 was also associated with reduced IR. This relationship was not observed in patients with other genotypes. The result is consistent with previous research, which showed that successful viral clearance was found to be associated with reduced HOMA-IR in patients with HCV genotype 1 (32). The genotype-specific outcome of IR suggested the direct viral effect as an important contributor to glucose abnormalities. In our result, advanced hepatic fibrosis was independently associated with IR. However, we did not observe a significant improvement in IR after HCV eradication in patients with advanced fibrosis. Liver fibrosis is a chronic process. Patients who received DAA therapy could have a rapid decrease in inflammation rather than regression of fibrosis. The duration of our study seemed too short for significant hepatocyte remodeling or IR improvement.

As discussed above, patients with chronic HCV infection show an increased risk of IR and progression to type 2 DM. There are no definite virus-specific factors associated with the increasing prevalence of IR. Thus, we suggest that all patients with HCV infection be screened for glucose abnormalities. It will help physicians to identify patients who have a higher risk of developing type 2 diabetes and may require active surveillance, as diabetes can also increase the risk of cirrhosis and hepatocellular carcinoma (33, 34). With the advent of DAAs, we may be able to eradicate HCV with greater certainty and a lower risk of adverse effects. An SVR is generally associated with normalization of liver enzymes, improvement in liver function, as well as regression of liver necroinflammation and fibrosis (35). Compared with untreated patients, patients whose HCV was eliminated by DAAs have a significantly lower risk of developing hepatocellular carcinoma (36). Furthermore, after successful virus elimination by treatment with DAAs, improvement in IR can be an additional benefit and may reduce diabetes-related morbidities. A prospective study found that HCV eradication by DAAs reduces the incidence of type 2 DM (37). However, a general worsening of the lipid profile after DAAs was observed (38, 39). The impact of the balance between improved glucose metabolism and negative changes in lipid profile on the outcome of cardiovascular and cardiometabolic risk must be determined. A significant reduction in major adverse cardiovascular events has recently been observed following HCV clearance by DAAs (40, 41). Further research is required to fully understand the underlying mechanisms.

This study has some limitations. First, this was an observational, open-label study and had the potential for selection bias. Second, a majority of the patients (98.0%) achieved SVR; therefore, it was impossible to compare glucose outcomes between patients with successful eradication and those with treatment failure. Third, the short-term study failed to demonstrate an improvement in IR; thus, the long-term outcome should be investigated to understand the effect of virus elimination on glucose homeostasis. Finally, we could not clarify the impact of HCV infection duration on IR, and could only demonstrate that treatment experience and advanced hepatic fibrosis were associated with IR.

In conclusion, we showed that obesity, treatment experience, baseline elevated levels of ALT and triglyceride, as well as advanced hepatic fibrosis were independently associated with high IR in chronic HCV-infected patients. The prevalence of IR was not influenced by HCV genotypes and viral load. This study provided evidence that HCV-related IR could be partially reversed by HCV eradication, especially in patients with high baseline IR, HCV genotype 1, and treatment experience. Further research for long-term outcomes of glucose homeostasis and diabetes-related morbidities in chronic HCV patients receiving DAAs is required.

## Data Availability Statement

The raw data supporting the conclusions of this article will be made available by the authors, without undue reservation.

## Ethics Statement

The studies involving human participants were reviewed and approved by Institutional Review Board of Mackay Memorial Hospital. The patients/participants provided their written informed consent to participate in this study.

## Author Contributions

C-HC: Design of the work, Writing. C-YC: Investigation, Supervision. H-LC: Interpretation of data, Review & Editing. I-TL: Analysis of data. C-HW: Methodology, Analysis of data. Y-KL: Design of the work, Review & Editing. M-JB: Resources, Supervision. All authors have seen the contents of the manuscript and agree to be accountable for all aspects of the work in ensuring that questions related to the accuracy or integrity of any part of the work are appropriately investigated and resolved. All authors contributed to the article and approved the submitted version.

## Conflict of Interest

The authors declare that the research was conducted in the absence of any commercial or financial relationships that could be construed as a potential conflict of interest.

## Publisher’s Note

All claims expressed in this article are solely those of the authors and do not necessarily represent those of their affiliated organizations, or those of the publisher, the editors and the reviewers. Any product that may be evaluated in this article, or claim that may be made by its manufacturer, is not guaranteed or endorsed by the publisher.
